# Tumor-Specific D-Dimer Concentration Ranges and Influencing Factors: A Cross-Sectional Study

**DOI:** 10.1371/journal.pone.0165390

**Published:** 2016-11-11

**Authors:** Jing Yu, Dongqing Li, Dansheng Lei, Feng Yuan, Feng Pei, Huifeng Zhang, Anming Yu, Kun Wang, Hu Chen, Liang Chen, Xianglei Wu, Xianli Tong, Yefu Wang

**Affiliations:** 1 The State Key Laboratory of Virology, College of Life Sciences, Wuhan University, Wuhan, Hubei 430072, China; 2 Department of Laboratory, Hubei Cancer Hospital, Wuhan, China; 3 Department of Microbiology, School of Basic Medical Sciences, Wuhan University; Wuhan, China; 4 Stago Diagnosis Company, Wuhan, China; 5 Department of Orthopaedics, Renmin Hospital, Wuhan University, Wuhan, China; 6 Laboratory of Immunology, University of Lorraine, Lorraine, France; University of Oklahoma Health Sciences Center, UNITED STATES

## Abstract

D-dimer level in cancer patients is associated with risk of venous thromboembolism and deep venous thrombosis. Most cancer patients have “abnormal” D-dimer levels based on the current normal reference range. To investigate tumor-specific D-dimer reference range, we compared D-dimer levels for nine different tumour types with healthy controls by using simultaneous quantile regression and constructing a median, 5th percentile, and 95th percentile model of normal tumour D-dimer concentration. Associations with tumour primary site, stage, pathological type, and treatment were also explored. Additionally, 190 patients were tracked to reveal the relevance of initial D-dimer levels to cancer prognosis. D-dimer ranges (median, 5th, 95th) in various cancers (mg/L) were: liver 1.12, 0.27, 5.25; pancreatic 0.96, 0.23, 4.81; breast 0.44, 0.2, 2.17; gastric 0.65, 0.22, 5.03; colorectal 0.73, 0.22, 4.45; lung 0.7, 0.25, 4.0; gynaecological 0.61, 0.22, 3.98; oesophageal 0.23, 0.7, 3.45; and head and neck 0.22, 0.44, 2.19. All were significantly higher than that of healthy controls (0.18, 0.07, 0.57). D-dimer peaked 1–2 days postoperatively but had decreased to the normal range by 1 week. Additionally, cancer patients with high initial D-dimer were shown a tendency of poor prognosis in survival rate. In conclusion, D-dimer levels in cancer depend on patient age, tumour primary site, and tumour stage. Thrombosis prevention is necessary if D-dimer has not decreased to the tumor-specific baseline a week after surgery.

## Introduction

D-dimer is a degradation product of crosslinked fibrin that appears in the blood after a blood clot is degraded by fibrinolysis [1]. Elevated D-dimer levels in the blood predict increased secondary fibrinolytic activity and are a principal marker of hypercoagulation and thrombosis [2–6]. Many studies have shown that high D-dimer levels are associated with the risk of deep venous thrombosis (DVT) and pulmonary embolism [7, 8], which are serious post-surgery complications with high mortality especially in patients with malignant tumours. Due to the injury to vascular endothelial cells caused by toxins released from fast growing tumour cells and the fibrinolytic activator on the surface of tumour cells, cancer patients often exhibit abnormal coagulation and fibrinolytic activities and their D-dimer levels tend to be higher than those in non-neoplastic populations. The D-dimer levels of almost all cancer patients exceed the recommended limits according to the existing reference range (0–0.5 mg/L). Therefore, the high risk of DVT according to this range might be overestimated [9–11].

It is suggested that the current D-dimer reference range is unsuitable for cancer patients, which limits the application of D-dimer testing in laboratory diagnosis and the prevention of tumour venous thrombosis and venous thromboembolism (VTE) [9–11]. The present prospective study measured plasma D-dimer levels in a large sample of cancer patients to investigate the range of D-dimer concentration in the absence of VTE. In addition, changes in D-dimer levels in cancer patients were determined during the perioperative period [9, 11] and after chemotherapy or radiotherapy.

## Materials and Methods

### Patients and controls

#### Patients with malignant tumours

A total of 1368 patients treated at Hubei Tumor Hospital and Wuhan Tongji Hospital between January, 2010 and August, 2013 were selected. The exclusion criteria comprised the following factors that may affect D-dimer level [12]: hypertension; diabetes; personal or family history of thromboembolic disease; coagulopathy; cardiovascular/cerebrovascular disease; autoimmune disease (such as rheumatoid arthritis, systemic lupus erythematosus, or idiopathic thrombocytopenic purpura); infection in the previous 30 days with body temperature > 37.5°C; surgery or trauma in the previous 30 days; blood transfusion in the previous half year; stressful state on the day before blood collection; and patient taking medications that may affect coagulation and the fibrinolytic system. Each patient was diagnosed with malignant disease, most based on pathology findings; some patients had advanced stage inoperable cancer confirmed with CT or another imaging method. The patients comprised the following: 142 liver cancer, 150 pancreatic cancer, 140 breast cancer, 120 stomach cancer, 120 colorectal cancer, 240 lung cancer, 120 gynaecological tumour, 123 oesophageal cancer, and 120 head and neck tumour. The average age was 55 years; 621 patients were male and 654 were female. Thirteen of the cancer patients (all male) were diagnosed with DVT and excluded from the study.

#### Patients with benign tumours

A total of 93 in-patients treated at Hubei Tumor Hospital from January, 2010 to August, 2013 were selected. The selection criteria were the same as above. Each patient received a pathological diagnosis of benign tumour. Their average age was 48.6 years; 20 were male and 73 were female.

#### Healthy control group

A total of 150 in-patients treated at Wuhan Tongji Hospital during the same period were selected. Their average age was 44.2 years; 103 were male and 47 were female.

#### Ethics Statement

This study was approved by the ethics committee of Hubei Tumor Hospital and Wuhan Tongji Hospital. All participants joined the study voluntarily and provided written informed consents. The committee approved the experiments and the methods were conducted in accordance with the approved guidelines.

### Methods

#### Sample collection, instruments, and reagents

Samples of 1.8 mL of elbow venous blood were collected in anticoagulant tubes with 109 mol/L sodium citrate for an anticoagulant to venous blood ratio of 1:9. After immediate mixing, the samples were centrifuged at 3000 × *g* for 10 min and then tested within 2 hours. D-dimer, activated partial thromboplastin time (APTT), prothrombin time (PT), thrombin time (TT), and fibrinogen (FiB) level were determined using a STA-R Evolution coagulant analyser with its specific reagents (Stago, Asnières-sur-Seine, France). Platelet levels were tested using a five-part blood cell counter with its reagents (Sysmex, Kobe, Japan).

#### D-dimer, APTT, PT, TT, and Fib assay

A STA-R Evolution coagulant analyser (Stago) was used to measure D-dimer, APTT, PT, TT, and FiB by immune turbidimetry. All samples were handled as routine clinical samples.

#### Analysing the correlation between D-dimer levels and tumour stages or tumour pathological types

D-dimer was compared among patients with tumours of stage I-II, or stage III-IV; moreover, in the same type of cancer, D-dimer was compared among patients with different pathological types.

#### D-dimer levels in tumour patients during the perioperative period

Of the 1275 patients with malignant tumours, 101 who underwent surgery were selected. These comprised 39 liver cancer, 10 breast cancer, 10 stomach cancer, 10 colorectal cancer, 10 lung cancer, 17 gynaecological tumour, and five head and neck tumour patients. Venous blood was collected on day 1, day 3, and 1 week after surgery for measurement of D-dimer level.

#### D-dimer levels in tumour patients before and after radio/chemotherapy

Of the 1275 patients with malignant tumours, 130 cases who underwent radio/chemotherapy were selected. These comprised 39 cases of liver cancer, 19 cases of breast cancer, 14 cases of stomach cancer, 18 cases of colorectal cancer, 35 cases of lung cancer, 2 oesophageal cancer, and 3 cases of gynaecological tumour patients. Of these 130 patients, 29 cases were treated with radiotherapy and 101 were treated with chemotherapy (detailed chemotherapy regiments see supporting information 1). All plasma samples were collected after one cycle of therapy to determine D-dimer levels.

#### Analysing the correlation between D-dimer levels and cancer prognosis

Of the 1275 patients with malignant tumours, 190 patients were tracked after being discharged from hospital to reveal the relevance of initial D-dimer levels to cancer prognosis. These cases comprised 15 cases of gastric cancer, 26 cases of colorectal cancer, 23 cases of liver cancer, 10 cases of pancreatic cancer, 30 cases of lung cancer, 15 cases of gynaecologic cancer, 48 cases of breast cancer, 21 cases of head neck cancer and 2 cases of oesophageal cancer.

### Statistical analysis

SPSS16.0 (IBM, Armonk, NY, USA) was used for statistical analysis. First, the Kolmogorov–Smirnov method was used to test the normality of the D-dimer data, which were found to have a non-normal distribution. Therefore, simultaneous quantile regression was performed to construct a median, 5th percentile, and 95th percentile model of normal tumour D-dimer concentration. The D-dimer levels of the benign tumour group, the malignant tumour group, and the healthy controls were compared using the Mann–Whitney *U* test; *P* < 0.05 indicated statistically significant differences. Comparisons between malignant tumours in different locations or the same malignant tumour at different stages or of different pathological types were performed using the Mann–Whitney *U* test; *P* < 0.05 indicated statistically significant differences. D-dimer levels before and after treatment were also compared using the Mann–Whitney *U* test, with *P* < 0.05 indicating statistically significant differences.

## Results

### D-dimer levels in benign tumour group, malignant tumour groups, and healthy controls

**Fig 1:**
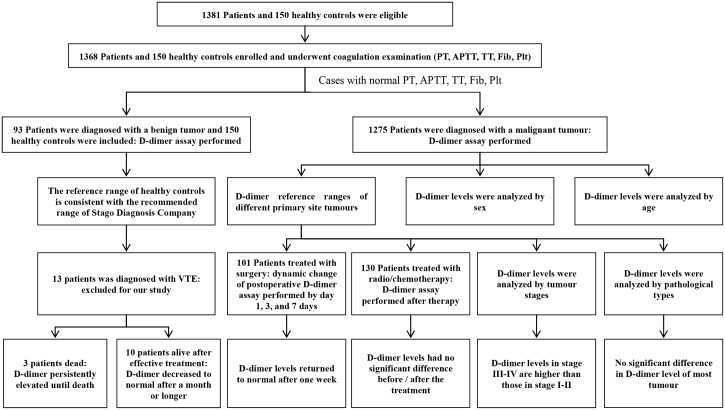
The diagram of the entire study. Enrolment and outcome.

Fig 1 displays a diagram of the entire study. The baseline characteristics of the patients are reported in Table 1. The conventional coagulation measures (APTT, PT, TT, Fib, and platelet count) in all cases were normal. The D-dimer level in each malignant tumour group was higher than that in the benign tumor group (*P* < 0.05) and in the healthy control group (*P* < 0.05). There was no significant difference between the benign tumour group and the healthy controls (*P* = 0.11). D-dimer levels in participants who were older than 55 years were much higher than those in patients less than 55 years old in the healthy controls and the benign tumour group, independent of gender. In the malignant tumour group, participants over 65 years had higher D-dimer levels than those below 65 years, independent of gender. Thus, gender did not affect the D-dimer level, whereas age did. The D-dimer levels of older participants were higher than those of younger participants.

**Table 1:** Baseline characteristics of the 1368 study patients and 150 healthy persons.

Characteristic	N Number	D-dimer level*	*P* value^§^	APTT(s)^#^	PT(s)^#^	TT(s)^#^	FiB(g/l)^#^	PLT(g/l)
**Healthy**	**150**	**0.25±0.29**		**36.0±3.16**	**13.0±0.52**	**16.4±0.68**	**2.9±0.69**	**182.5±51.76**
**Sex**								
** Female**	**47**	**0.26±0.19**	**0.151(F/M)**	**35.8±3.35**	**13.0±0.54**	**16.2±0.78**	**3.0±0.55**	**176.0±57.41**
** Male**	**103**	**0.20±0.17**		**36.1±3.08**	**13.0±0.51**	**16.4±0.62**	**2.9±0.75**	**185.5±48.98**
** Age (years)**								
** Mean**	**43.6**							
**<55—no. (%)**	**121 (81.3)**	**0.26±0.21**	**0.007 (Y/O)**	**36.1±3.19**	**13.0±0.51**	**16.3±0.7**	**2.8±0.69**	**186.4±57.33**
**≥55—no. (%)**	**28 (18.67)**	**0.30±0.19**		**35.8±3.06**	**13.0±0.57**	**16.6±0.55**	**3.1±0.66**	**176.9±50.67**
**Benign tumour**	**93**	**0.32±0.14**	**0.11(B/H)**	**35.6±1.85**	**12.6±0.57**	**17.7±1.15**	**2.8±0.46**	**182.9±56.85**
**Sex**								
** Female**	**73**	**0.25±0.04**	**0.422 (F/M)**	**35.6±1.76**	**12.5±0.54**	**17.7±1.1**	**2.8±0.46**	**182.1±54.07**
** Male**	**20**	**0.30±0.13**		**35.8±2.19**	**12.7±0.65**	**17.8±1.35**	**2.8±0.49**	**186.0±67.50**
**Age (years)**								
** Mean**	**48.6**							
**<55—no. (%)**	**65(69.9)**	**0.25±0.05**	**0.000 (Y/O)**	**35.6±1.69**	**12.5±1.56**	**17.7±1.19**	**2.7±0.43**	**177.1±56.32**
**≥55—no. (%)**	**28 (30.1)**	**0.43±0.17**		**35.8±2.2**	**12.8±0.58**	**17.9±1.05**	**3.0±0.48**	**196.5±56.72**
**Malignant tumour**	**1275**			**36.3±2.12**	**12.6±0.61**	**17.5±1.24**	**2.9±0.50**	**194.4±47.00**
**Sex**								
** Female**	**621**	**1.20±1.50**	**0.055 (F/M)**	**35.9±1.78**	**12.7±1.05**	**16.8±1.04**	**2.9±0.45**	**192.9±45.87**
** Male**	**654**	**1.07±1.04**		**36.1±2.35**	**12.8±0.65**	**16.7±0.86**	**2.8±0.52**	**196.7±47.19**
**Age (years)**								
** Mean**	**55.0**							
**<65 -no. (%)**	**1097(85.16)**	**1.23±1.55**	**0.013 (Y/O)**	**35.7±3.02**	**12.8±0.74**	**17.8±0.56**	**2.9±0.45**	**192.1±41.16**
**≥65 -no. (%)**	**188(14.84)**	**1.47±1.72**		**35.6±2.86**	**12.7±0.62**	**17.4±0.75**	**3.0±0.56**	**207.2±70.84**

*D-dimer levels are shown as the mean ± standard deviation (SD) (μg/mL).

^§^*P* values were calculated using the Mann–Whitney *U* test; *P* < 0.05 indicates that the difference was statistically significant. F/M represents D-dimer levels of females vs. males; Y/O represents younger vs. older patients. B/H refers to the D-dimer levels of benign tumour patients compared with those of healthy controls.

^#^Reference ranges of APTT, PT, TT, and Fib are 32–40 s, 12–14 s, 14–21 s, and 2–4 g/L, respectively. These values are also expressed as the mean ± SD.

### D-dimer range and level in tumour patients and healthy controls

**Fig 2:**
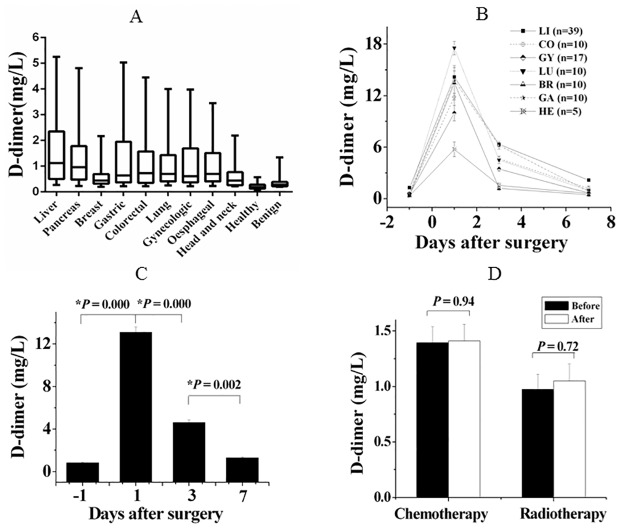
Tumor-specific D-dimer levels and influencing factors. **(A)** Box diagram of D-dimer levels in cancer patients and healthy controls. Displayed on this diagram are the median, 5th centile, and 95th centile concentrations (solid lines; 5%, 50%, and 95% from bottom to top). **(B)** Change in D-dimer level in tumour patients with day after surgery. Details of the 101 malignant tumour patients and the procedure for D-dimer measurement are described in the Materials and Methods. The y-axis shows the D-dimer concentration (mg/L). Data were expressed as the mean relative concentration (mg/L) ± the standard error of the mean (SEM). The x-axis shows the number of days after surgery. LI, liver cancer; CO, colorectal cancer; GY, gynaecological tumour; LU, lung cancer; BR, breast cancer; GA, gastric cancer; HE, head and neck cancer. **(C)** D-dimer levels 1 day, 3 days, and 1 week after surgery compared with that before surgery; P < 0.05 indicates that the difference was statistically significant. **(D)** The effects of radio/chemotherapy on plasma D-dimer level. D-dimer levels before and after radio/chemotherapy were compared. Plasma samples were collected after one cycle of the therapy. Details of the 130 malignant tumour patients and the procedure for D-dimer measurement are described in the Materials and Methods. The y-axis represents D-dimer level (mg/L). Data were expressed as the mean relative concentration (mg/L) ± SEM. The x-axis represents before and after radio/chemotherapy. P < 0.05 indicates that the difference was statistically significant.

The ranges of D-dimer concentration in malignant and benign tumour patients and healthy controls were represented as the median and 5th and 95th percentile values (Fig 2A). D-dimer levels in liver, pancreatic, stomach, colorectal, lung, and oesophageal cancer patients were significantly higher than those in breast cancer patients (*P* < 0.05) (Table 2, Fig 2A).

**Table 2:** D-dimer levels (mg/L) for each malignancy and for healthy controls.

Group [n; median (mg/L); SE]	Mann-Whitney *U*-tests for each group versus healthy controls [n = 150; median (mg/L) = 0.18; SE = 0.024]; Other cancer groups versus breast cancer			
	W	Z-score	*P(Group Vs. healthy control)	*P (Group VS. Breast cancer)
**Liver cancer (142, 1.12, 0.144)**	**12416.**	**-13.257**	**0.000**	**0.000**
**Pancreatic cancer (150,0.96, 0.121)**	**12814**	**-12.995**	**0.000**	**0.000**
**Breast cancer (140,0.44,0.056)**	**14009.5**	**-10.955**	**0.000**	
**Gastric cancer (120, 0.65, 0.187)**	**12892.5**	**-11.660**	**0.000**	**0.000**
**Colorectal cancer (120, 0.73, 0.135)**	**13240.5**	**-11.114**	**0.000**	**0.000**
**Lung cancer (240, 0.7, 0.089)**	**14053.5**	**-14.102**	**0.000**	**0.000**
**Gynecologic cancer (120, 0.61, 0.125)**	**12916.5**	**-11.622**	**0.000**	**0.000**
**Oesophageal cancer (123, 0.7, 0.108)**	**12897.0**	**-11.793**	**0.000**	**0.000**
**Head neck tumour (120, 0.44, 0.165)**	**13841.5**	**-10.172**	**0.000**	**0.716**

*Differences were considered significant when *P* < 0.05.

### D-dimer level and tumour stage

When D-dimer was compared among patients with tumours of differing stage in the same primary site, those with stage III/IV disease had significantly higher levels than those with stage I/II disease (*P* < 0.05), as shown in Table 3.

**Table 3:** D-dimer levels (mg/L) for different stages of each malignancy.

Group	Stage	D-dimer level*	^§^*P* value
		[n, median(mg/L), SE,]	(III-IV VS I-II)
**Liver cancer**	**I-II**	**21, 0.49, 0.136**	**0.000**
	**III-IV**	**121, 1.29, 0.208**	
**Pancreatic cancer**	**I-II**	**21, 0.52, 0.082**	**0.000**
	**III-IV**	**129, 1.06, 0.136**	
**Breast cancer**	**I-II**	**57, 0.35, 0.052**	**0.000**
	**III-IV**	**83, 0.52, 0.157**	
**Gastric cancer**	**I-II**	**20, 0.42, 0.067**	**0.000**
	**III-IV**	**100, 0.92, 0.216**	
**Colorectal cancer**	**I-II**	**22, 0.42, 0.106**	**0.002**
	**III-IV**	**98, 0.82, 0.159**	
**Lung cancer**	**I-II**	**45, 0.40, 0.078**	**0.000**
	**III-IV**	**195, 0.82, 0.106**	
**Gynecologic cancer**	**I-II**	**56, 0.42, 0.090**	**0.000**
	**III-IV**	**64, 1.20, 0.204**	
**Oesophageal cancer**	**I-II**	**25, 0.46, 0.153**	**0.007**
	**III-IV**	**98, 0.80, 0.129**	
**Head neck cancer**	**I-II**	**42, 0.32, 0.044**	**0.000**
	**III-IV**	**78, 0.55, 0.248**	

*D-dimer levels are expressed as the median (mg/L), standard error (SE).

^§^*P* values were calculated using the Mann–Whitney *U* test; *P* < 0.05 indicates that the difference was statistically significant.

### D-dimer level and pathological type

When D-dimer was compared among patients with cancer in the same primary site but of differing pathological type, there were no significant differences for most tumour types (*P* > 0.05). D-dimer levels were higher in bile duct cell carcinoma patients than in hepatocellular carcinoma patients (*P* = 0.018) and in squamous cell carcinoma compared with adenocarcinoma in patients with lung cancer (*P* = 0.035) (Table 4).

**Table 4:** D-dimer levels for different tumor pathological types.

Group	^#^Pathological types	D-dimer level [n, median(mg/L), SE]	*P value
**Liver cancer**	**H**	**45, 0.62, 0.142**	**0.018 (H/B)**
	**B**	**23, 1.94, 0.407**	
**Pancreatic cancer#**	**A**	**54, 1.08, 0.230**	
**Breast cancer**	**L**	**13, 0.50, 0.258**	**0.770 (L/D)**
	**D**	**127, 0.44, 0.056**	
**Gastric cancer#**	**A**	**67, 1.33, 0.224**	
**Colorectal cancer#**	**A**	**46, 1.06, 0.177**	
**Lung cancer**	**S**	**48, 1.06, 0.305**	**0.035(S/A)**
	**A**	**148, 0.68, 0.136**	**0.698(A/SC)**
	**Sc**	**38, 1.01, 0.829**	**0.294(S/SC)**
**Gynecologic cancer**	**S**	**73, 0.57, 0.114**	**0.367 (S/A)**
	**A**	**28, 0.68, 0.281**	
**Oesophageal cancer#**	**S**	**89, 0.75, 0.073**	
**Head neck cancer**	**S**	**80, 0.45, 0.241**	**0.137(S/A)**
	**A**	**31, 0.35, 0.146**	

S, squamous cell carcinoma; A, adenocarcinoma; H, hepatocellular carcinoma; B, bile duct cell carcinoma; L, infiltrating lobular carcinoma; D, infiltrating ductal carcinoma; SC, small cell lung cancer.

*P values were calculated using the Kruskal–Wallis *H* test; *P* < 0.05 indicates that the difference was statistically significant. H/B represents D-dimer levels of hepatocellular carcinoma patients vs those of bile duct cell carcinoma patients. L/D, infiltrating lobular carcinoma vs. infiltrating ductal carcinoma; S/A, squamous cell carcinoma vs. adenocarcinoma;A/SC, adenocarcinoma vs. small cell lung cancer; S/SC, squamous cell carcinoma vs adenocarcinoma.

^#^Only a single type was collected in our study despite there being several pathological types of the disease.

### Changes in D-dimer levels of tumour patients without thrombosis in the perioperative period

**Fig 3:**
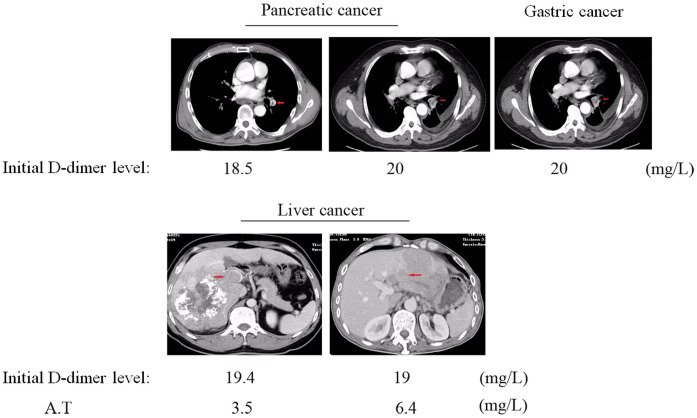
CT films of cancer patients with VTE. A.T means after treatment. Red errows indicate the VTE sites.

Plasma D-dimer levels in 101 tumour patients without thrombosis were significantly raised (up to 20 mg/L) on day 1 after surgery but had significantly decreased on day 3; they had returned to within the recommended range for cancer patients 1 week after surgery (Fig 2B and 2C). In some cases, D-dimer levels continued to increase after surgery with no sharp decrease after 1 week; this was observed in one stomach cancer patient, ten liver cancer patients, and two pancreatic cancer patients. Five of them had definitely confirmed VTE by CT films (Fig 3). Ten of them recovered and three died. Continued monitoring of the 10 recovered patients showed that their D-dimer levels gradually decreased to within the recommended range for cancer.

### Effect of radio/chemotherapy on plasma D-dimer levels

There was no significant difference between plasma D-dimer levels before and after treatment in 101 patients who underwent chemotherapy and 29 who received radiotherapy (*P* = 0.94 and *P* = 0.72, respectively) (Fig 2D).

### Initial D-dimer levels correlated with prognosis

Survival rate of some cancer patients were successfully tracked by the end of 2015. As shown in Table 5, cancer patients with higher initial D-dimer value were shown poor prognosis compare with those who had lower initial D-dimer value.

**Table 5:** Initial D-dimer level were associated with a poor prognosis.

Group	Numbers of death	Initial D-dimer level [median (mg/L), SE]	Numbers of survival	Initial D-dimer level [median (mg/L), SE]	Survival rate by the end of 2015 (%)	*P* value
**Gastric cancer**	**13**	**0.66, 0.41**	**2**	**0.35, 0.095**	**13.33**	
**Colorectal cancer**	**18**	**1.21, 1.16**	**8**	**0.25, 0.09**	**30.77**	
**Liver cancer**	**19**	**1.68, 0.46**	**4**	**0.40, 0.05**	**17.39**	
**Pancreatic cancer**	**10**	**1.12, 0.30**	**0**	**0**	**0.00**	
**Lung cancer**	**21**	**1.03, 0.98**	**9**	**0.41, 0.12**	**30.00**	
**Gynecologic cancer**	**5**	**1.90, 0.45**	**10**	**0.30, 0.04**	**66.67**	
**Breast cancer**	**0**	**0**	**48**	**0.36, 0.04**	**100.00**	
**Head neck tumor**	**0**	**0**	**21**	**0.31, 0.09**	**100.00**	
**Oesophageal cancer**	**0**	**0**	**2**	**0.35, 0.22**	**100.00**	
**Total**	**86**	**1.27,0.36**	**104**	**0.36, 0.03**	**54.74**	**0.000**

*P value: Initial D-dimer levels in all dead patients’ plasma vs. those in survival patients’ plasma. *P* values were calculated using the Mann–Whitney *U* test; *P* < 0.05 indicates that the difference was statistically significant.

## Discussion

The kit used to measure D-dimer levels in this study is commonly employed in clinical laboratories and in its instructions defines a concentration of 0.5 mg/L as normal. This value has been shown to be appropriate for healthy people [13]; however, more than 60% of cancer patients with normal coagulation exhibit D-dimer levels greater than 0.5 mg/L. In the present study we aimed to investigate the range of D-dimer concentration in cancer patients and its influencing factors. Even if such a range does not contribute to the diagnosis of VTE, it can provide data to support the exclusion of this disease. All steps in the study strictly followed the rules set out in the CLSIC28-A3 document [14] and subjects were selected based on rational inclusion and exclusion criteria.

To explain the differences in D-dimer level among the various cancers, we systematically analysed all patient data, including their basic characteristics, routine clinical laboratory tests, clinical treatment, and pathological diagnosis. Broadly, in the cancer population D-dimer level was independent of gender but was affected by the age of the patient and the stage of the tumour. D-dimer was also influenced by the pathological type of the tumour in bile duct cell carcinoma patients, who had higher levels than hepatocellular carcinoma patients, and, among lung cancers, in squamous cell carcinoma patients, who had higher levels than adenocarcinoma patients (*P* < 0.05); these significant differences may have been due to the later stage of the cancers in these cases.

Haase *et al*. showed that D-dimer levels increased markedly with age in healthy individuals [15]. Consistent with this study, we found that patients aged over 55 years had higher D-dimer levels than those aged less than 55 years (*P* < 0.05). Haase *et al*. also reported a certain degree of differences were found between the reference ranges for males and females among older people; yet they also indicated the difference was minor and the clinical relevance was highly questionable. Therefore, no difference of D-dimer levels between the sexes in our study is a comprehensible result.

Interestingly, we found that the ranges of D-dimer concentration in breast cancer and head and neck tumour patients differed from those in the other malignancies. D-dimer levels in patients with breast or head and neck cancer were much lower than those in patients with liver, pancreatic, stomach, colorectal, lung, gynaecological, or oesophageal cancer (*P* < 0.05). Some studies have reported D-dimer levels in cancer patients to be strongly associated with the number of metastatic nodes and patient prognosis [16]. Compared with liver, pancreatic, stomach, colorectal, lung, gynaecological, and oesophageal cancer, breast and head and neck cancers have lower metastatic rates and a more favourable prognosis based on clinical statistics [17, 18]. Although the prevalence of lymph node metastasis might be high in the early stages of breast cancer, it is generally accepted that 5-year survival in breast cancer is much higher than that for other carcinomas. We also found that, among patients with the same cancer, D-dimer levels were markedly higher in stage III/IV disease than in stage I/II. The significant differences observed between tumour stages show that D-dimer level is also strongly associated with the grade of malignancy.

Surgery may induce embolisms [19]. Our findings demonstrate the importance of monitoring D-dimer levels in all perioperative cancer patients. D-dimer changes in such patients show a specific trend, as described above. Patients whose D-dimer changes resemble this trend may suffer VTE, and persistently elevated D-dimer levels may indicate a poor prognosis. It is therefore crucial to monitor the D-dimer levels of cancer patients at 3 days and 7 days postoperatively. Clinicians should take the necessary measures to prevent VTE or DVT if levels have not decreased to normal 1 week after surgery.

The D-dimer levels of 130 patients with malignant tumours were measured 1 week after they underwent radio/chemotherapy. D-dimer did not appear to be influenced by radio/chemotherapy based on our results. The use of bevacizumab combined with chemoradiotherapy is associated with a higher risk of VTE compared with antiangiogenic therapy alone [20, 21]. Among 29 patients in our study who underwent radiotherapy and 101 who received chemotherapy, none was given bevacizumab at any point in his or her treatment. Therefore, we suggest that the effects of radio/chemotherapy on D-dimer levels should be evaluated in light of any drugs that the patient has been administered.

The D-dimer level of patients whether can be served as an indicator of prognosis is also drawing us, even though so many factors might affect 5-year survival rate of cancer patients [22, 23]. As we expected, our study showed the initial D-dimer level of dead patients were obviously higher than that of the patients who were still alive at the end of 2015. Apart from gastric cancer patients, the initial d-dimer levels of other dead patients were higher than 1.00 mg/L, including patients in liver cancer, pancreatic cancer, lung cancer and gynaecologic cancer. These results consist with other reports before [24, 25, 26, 27, and 28]. The previous study with regard to lung cancer found the d-dimer median concentration was 0.84mg/L [29]. As presented in our results, such level was 0.7mg/L. To date, our study firstly analyzed the d-dimer ranges on the other types of cancer. However, we couldn’t make a conclusion on the definite d-dimer range which can be served as an indicator of poor prognosis, because the cases of tracked patients are not idea. Hence, further studies are required to confirm our findings.

## Conclusions

We have discussed the cancer-specific concentration range for D-dimer and evaluated factors that could influence D-dimer levels in cancer patients. D-dimer level is independent of gender but dependent on patient age, tumour primary site, and tumour stage. In addition, monitoring changes in D-dimer level is critical for all perioperative cancer patients. Clinicians should take the necessary measures to prevent VTE or DVT if the D-dimer level has not decreased to the recommended range 1 week after surgery. The applicability of this research should be further investigated in a large prospective study. When sufficient cases and influencing factors are available, we may be able to establish an acceptable “normal” D-dimer reference range for cancer patients.

## Supporting Information

S1 Table The chemotherapy regiments for 101 cancer patients. The detail chemotherapy regiments were used for different cancer patients.
(PDF)
