# Supplementary material for: Tumor-Specific D-Dimer Concentration Ranges and Influencing Factors: A Cross-Sectional Study
Source: PLoS One. 2016 Nov 11;11(11):e0165390. doi: 10.1371/journal.pone.0165390 (PMC5105993; doi:10.1371/journal.pone.0165390)
Supplement: S1 Table — The detail chemotherapy regiments were used for different cancer patients. (PDF) [file pone.0165390.s001.pdf]

S1 Table The chemotherapy regiments for 101 cancer patients

Group(n)	Chemotheraphy Regimen
Liver cancer(28)	Oxaliplatin+Calcium folinate +5-fluorouracil (FOLFOX)
Breast cancer(8)	Epirubicin+Cyclophosphamide+Docetaxel (AC-T)
Gastric cancer(10)	Oxaliplatin+Calcium folinate+5-fluorouracil (FOLFOX)
Colorectal cancer(15)	Irinotecan+Calcium folinate+5-fluorouracil (FOLFIRI)
Non-small cell lung cancer(20)	Paclitaxel+Cisplastin (TP)
Small lung cancer(15)	Etoposide+Cisplatin (EP)
Ovarian cancer(3)	Paclitaxel+Cisplastin (TP)
Oesophageal cancer(2)	Capecitabine+Cisplastin
